# NF-κB regulation in maternal immunity during normal and IUGR pregnancies

**DOI:** 10.1038/s41598-021-00430-3

**Published:** 2021-10-25

**Authors:** Gaayathri Ariyakumar, Jonathan M. Morris, Kelly J. McKelvey, Anthony W. Ashton, Sharon A. McCracken

**Affiliations:** 1 Division of Perinatal Medicine, Faculty of Medicine and Health, The University of Sydney, and Northern Sydney Local Health District Research (Kolling Institute), St Leonards, NSW 2065 Australia; 2 Bill Walsh Translational Cancer Research Laboratory, Faculty of Medicine and Health, The University of Sydney, and Northern Sydney Local Health District Research (Kolling Institute), St Leonards, NSW 2065 Australia; 3 Department of Obstetrics and Gynaecology, Royal North Shore Hospital, St Leonards, NSW 2065 Australia

**Keywords:** Lymphocytes, Immunology, Medical research, Biomarkers

## Abstract

Intrauterine Growth Restriction (IUGR) is a leading cause of perinatal death with no effective cure, affecting 5–10% pregnancies globally. Suppressed pro-inflammatory Th1/Th17 immunity is necessary for pregnancy success. However, in IUGR, the inflammatory response is enhanced and there is a limited understanding of the mechanisms that lead to this abnormality. Regulation of maternal T-cells during pregnancy is driven by Nuclear Factor Kappa B p65 (NF-κB p65), and we have previously shown that p65 degradation in maternal T-cells is induced by Fas activation. Placental exosomes expressing Fas ligand (FasL) have an immunomodulatory function during pregnancy. The aim of this study is to investigate the mechanism and source of NF-κB regulation required for successful pregnancy, and whether this is abrogated in IUGR. Using flow cytometry, we demonstrate that p65^+^ Th1/Th17 cells are reduced during normal pregnancy, but not during IUGR, and this phenotype is enforced when non-pregnant T-cells are cultured with normal maternal plasma. We also show that isolated exosomes from IUGR plasma have decreased FasL expression and are reduced in number compared to exosomes from normal pregnancies. In this study, we highlight a potential role for FasL^+^ exosomes to regulate NF-κB p65 in T-cells during pregnancy, and provide the first evidence that decreased exosome production may contribute to the dysregulation of p65 and inflammation underlying IUGR pathogenesis.

## Introduction

Intrauterine growth restriction (IUGR) is the second leading cause of fetal morbidity, second only to premature birth^1^. With no known therapy for IUGR, early delivery remains the only viable treatment for IUGR. However, premature delivery poses significant risks to the baby and predisposes to the development of chronic diseases in childhood and adulthood such as insulin resistance, obesity, cardiovascular and kidney disease^2–6^. The exact cause of IUGR remains unknown. However, understanding the role of maternal immune adaptation during pregnancy has important implications for developing novel strategies for the prevention of pathological conditions associated with pregnancy like IUGR^7–13^.

During normal pregnancy, there is a shift from a T-helper (Th)1 to Th2 bias which induces maternal immune tolerance and suppression^14^. In contrast, pregnancy complications such as recurrent spontaneous miscarriage and preeclampsia (PE) are associated with a milieu of increased Th1 cytokines, including Interleukin (IL)-6, IL-8, Interferon Gamma (IFN-γ) and Tumour Necrosis Factor Alpha (TNF-α)^15–18^, which may act as markers of pregnancy pathology^19^. Normal pregnancy also requires an increased immunosuppressive Treg/Th17 ratio, a phenomenon highlighted by the amelioration of autoimmune symptoms in late pregnancy of women with co-morbid Rheumatoid Arthritis and Multiple Sclerosis; diseases characterised by increased Th17 and diminished Tregs^20–22^. Akin to Th1/Th2, imbalances in the Th17/Treg subsets are reported in the pregnancy complications PE and recurrent spontaneous abortion^23,24^. Therefore, understanding the mechanisms that regulate the immune balance between T-cell subsets during normal pregnancy can help elucidate the immune profile during complications like IUGR.

Nuclear Factor Kappa B (NF-κB) plays an integral role for the regulation of T-cell development and differentiation into effector subsets. NF-κB is comprised of five sub-units (p50, p65/RelA, c-Rel, p52 and RelB), which are activated by various stimuli, resulting in the transcription of genes including cytokines, adhesion molecules, cell cycle regulators and cell survival factors. Abnormal NF-κB signaling has been associated with a number of human diseases, including cancer, chronic inflammatory diseases and autoimmune diseases such as RA and MS^25,26^. Limited in vitro and in vivo studies have given insight into the role of NF-κB throughout gestation, (reviewed in Sakowicz A. 2018^27^). As a result, the role of NF-κB during pregnancy in humans, is poorly defined. We have previously shown that the p65 subunit which regulates Th1 responses^28^ and the differentiation of Th17 cells^29^, is reduced in CD3^+^ T-cells throughout pregnancy^30^. It was also identified that the suppression of p65 in CD3^+^ T-cells reduced the Th1 transcription factor Tbet^31^, as well as decreasing Th1 IFN-γ, IL-2 and TNF-α cytokine production in response to phorbol myristate acetate (PMA) stimulation^32^. As NF-κB regulates the transcription of several genes involved in T-cell function, such as Th1 differentiation and clonal expansion^33^, and Th17 differentiation^34^, a reduction in p65 in T-cells during pregnancy may explain why there is a Th2/Treg bias in normal pregnancy. Given these results, alterations in p65 expression in individual T-cell subsets may be responsible for the changes in T-cell function observed during pregnancy. Equally, failure to downregulate NF-κB may contribute to the increased inflammatory T-cell responses associated with adverse pregnancy outcomes like IUGR.

While the mechanism that regulates maternal T-cell p65 suppression is poorly understood, the Fas/FasL signalling pathway can regulate suppression of p65 via caspase degradation^35,36^. Our previous data in Jurkat T-cells revealed that Fas activation degrades p65 and the T-cell receptor transmembrane protein CD3ζ, both of which are integral for T-cell survival and function^37^. In addition, it is reported that CD3ζ expression is lost in the presence of sera from pregnant patients, and membrane fragments isolated from the sera were FasL^+^^38^. Together, these implicate a role for plasma soluble factors in the regulation of maternal T-cell p65 expression and responses. During pregnancy, FasL^+^ microvesicles present in maternal plasma mediate apoptosis as a mechanism for immune protection^39^. Exosomes are a subtype of small secreted extracellular vesicles (EVs) (30–150 nm), known to possess biological functions that impact cellular communication, particularly in cancer progression^40^. Since the concentration of exosomes, increases throughout the gestation of pregnancy^41^ and placenta derived exosomes express FasL^42^, exosomes may play a role in maintaining pregnancy success. However, their precise mechanism of action in the progression of pregnancy complications is not completely understood.

In this study it is hypothesised that changes in maternal T-cell phenotypes necessary for pregnancy success are regulated by suppression of p65, and is potentially mediated by EVs present in maternal plasma.

## Materials and methods

### Sample cohort

Whole blood was collected with informed consent from healthy non-pregnant women (NP; n = 11), third trimester normal pregnant women (P; n = 12) and third trimester pregnant women with IUGR (IUGR, n = 8). Healthy NP women were of reproductive age (21–38 years) not taking oral contraception or synthetic hormones, and whole blood collected during the pre-ovulatory phase of their menstrual cycle (days 7–14 post-menstruation). Normal third trimester pregnancies were defined as pregnant women unaffected by PE, IUGR or any other significant medical disorders and not on any form of medication. IUGR pregnancies were defined as pregnancies unaffected by preeclampsia with a birth weight < 10th percentile for gestational age, according to gender-specific data adjusted to national birth weight percentiles^43^, accompanied by an abnormal umbilical artery Doppler velocity flow defined as absent or reverse end diastolic velocity. 2/8 women with IUGR were on corticosteroids (Betamethasone). All pregnant women recruited for this study were nulliparous. This study was approved by the Northern Sydney Local Health District Human Research Ethics Committee at Royal North Shore Hospital, Sydney, Australia (protocol number: 1201-046 M). All experiments were performed in accordance with the relevant guidelines and regulations.

### Isolation of PBMCs, plasma and exosomes

PBMCs were isolated from blood collected in lithium heparin tubes by Ficoll-Paque density centrifugation, from NP, P and IUGR pregnant women for assessment by flow cytometry. Plasma samples were obtained by centrifugation at 389 × *g* for 15 min at 22 °C, without applied deceleration. Plasma was used for cell culture with PBMCs for analysis by flow cytometry, as well as assessment of EV concentration by NanoSight. Exosomes were isolated from the plasma by size exclusion chromatography (SEC) columns (iZon Science, New Zealand), according to the manufacturer’s protocol. Briefly, plasma was thawed on ice and centrifuged at 2,000 × *g* for 20 min, followed by 12,000 × *g* for 45 min at 4 °C to remove cellular debris. Columns were washed with PBS, and the flow rate was determined as a measure of column cleanliness. Subsequently, 0.5 mL of plasma was added to the top of the column, and topped with PBS, to prevent the filter from drying out. Multiple 0.5 mL fractions of the resulting elution were collected in 1.5 mL microcentrifuge tubes, as the constituents separated through a Sepharose matrix. Fractions were collected individually and measured for concentration and purity based on size distribution, as determined by NanoSight and Nanoparticle Tracking Analysis (NTA).

### Identification of plasma derived vesicles by nanoparticle tracking analysis (NTA)

To identify the number of vesicles and their size in plasma, plasma samples diluted in PBS (1:10,000), were subjected to NTA using the NanoSight NS300 (Malvern Instruments, UK) following the manufacturer’s instructions. Three 60 s videos were taken under automated settings to determine particles per mL and mean particle diameter. Particle concentration and size distribution were automatically analysed with the NanoSight Tracking Analysis Software Version 3.2.

### Cell culture and stimulation

PBMCs isolated from NP, P and IUGR women were cultured at 0.5 × 10^6^ cells/ml in RPMI-1640 media containing phenol, 10% (v/v) heat inactivated fetal calf serum (FCS), 100 IU/mL Penicillin and 100 ug/mL Streptomycin. Cells were cultured at 37 °C in a humidified incubator containing 5% (v/v) CO_2_. To compare the percentage of T-cells expressing subset specific cytokines, isolated PBMCs were stimulated with 50 ng/mL PMA and 500 ng/mL ionomycin in the presence of 10 ng/mL Brefeldin A for 4 h at 37 °C and assessed by flow cytometry. To assess differences in T-cell subsets expressing p65, isolated PBMCs were analysed by flow cytometry immediately following PBMC isolation.

### Cell staining for flow cytometry

For assessment of cytokines and p65 in T-cell subsets by flow cytometry, monoclonal antibodies and respective isotype controls were titrated to determine optimal staining concentration (Supplementary Table 1). Cells were centrifuged, supernatant discarded, and resuspended in Fluorescence Activated Cell Sorting (FACS) buffer (PBS and 0.1% BSA) containing the appropriate cell surface antibody or isotype control using BD Pharmingen Abs: anti-CD4/8 FITC or anti-CD95 PE-Cy5. Cells were incubated at 4 °C in the dark, for 30 min, and washed with FACS buffer afterwards. To preserve staining, the cells were fixed using FACS fix solution (PBS containing 0.1% BSA and 1% PFA). Following fixation, cells were washed with FACS buffer, and resuspended in FACS permeabilisation buffer (PBS containing 0.1% BSA and 0.1% saponin). Following permeabilisation, cells were washed and resuspended in FACS permeabilisation buffer, where appropriate antibodies for intracellular proteins were added to the cells: anti-Tbet/GATA3/RORγt/FOXP3 Alexa Fluor 647; anti-IL-2/IFN-γ/IL-4/IL-17/TGF-β PE (all from BD Pharmingen); or anti-NF-κBp65 PE (BioLegend). Cells were incubated in the dark for 45 min at 4 °C, and subsequently washed with FACS perm buffer, and resulting pellets were resuspended in FACS buffer for analysis by flow cytometry.

### Flow cytometry

Flow cytometry was performed with a FACSCalibur Flow Cytometer, using FlowJo software version 10 (Becton Dickinson). Approximately 10,000 lymphocyte events per sample were acquired for analysis. Lymphocytes were identified within PBMCs according to their size and granularity, as determined by forward and side scatter plots respectively. After gating for CD4^+^ or CD8^+^ T-cells from the lymphocyte population, percentage of cells expressing intracellular proteins (cytokine or p65) were determined by setting quadrants, using unstimulated cells as a biological negative control for cytokine analysis, or the relative isotype control from uncultured cells for p65 analysis. From this gated population, T-cell subsets were delineated using transcription factor positivity.

### Western blotting

Isolated exosome pellet was lysed in Radio-Immunoprecipitation Assay (RIPA) buffer and subjected to western blotting. Protein samples were separated on 10% (w/v) SDS–polyacrylamide gels and transferred to nitrocellulose membranes which were blocked as previously described^30^. Blots were probed with primary antibodies (Supplementary Table 2) against Tumour susceptibly gene 101 (TSG101) (1:500, Santa Cruz) and FasL (1:1000, Santa Cruz). GAPDH (1:1000, Bio-Rad) served as the loading control. Antibody binding was detected by specific secondary antibodies conjugated to HRP (1:1000, Bio-Rad), and to visualise protein bands, the Luminata Classico Western HRP Substrate (Merck-Millipore) was used. Pre-mixed reagent was used per blot and proteins were visualised using the ImageQuant LAS-4000 luminescent image analyser (GE Healthcare, Australia). Densitometry analysis was performed using the Image-J digital software version 1.8.0 with bands of interest standardised against Glyceraldehyde 3-phosphate dehydrogenase (GAPDH).

### Statistical analysis

For comparisons between pregnancy cohorts, the non-parametric Kruskal–Wallis with Dunn’s multiple comparisons test was used due to small sample size (n = 8–12). All data are presented as median and interquartile range (IQR; Q1 = 25th and Q3 = 75th percentile. Statistical significance was accepted when the *p* value was < 0.05.

## Results

### Th1 and Th17 cytokine production are altered in lymphocytes during pregnancy

**Figure 1:**
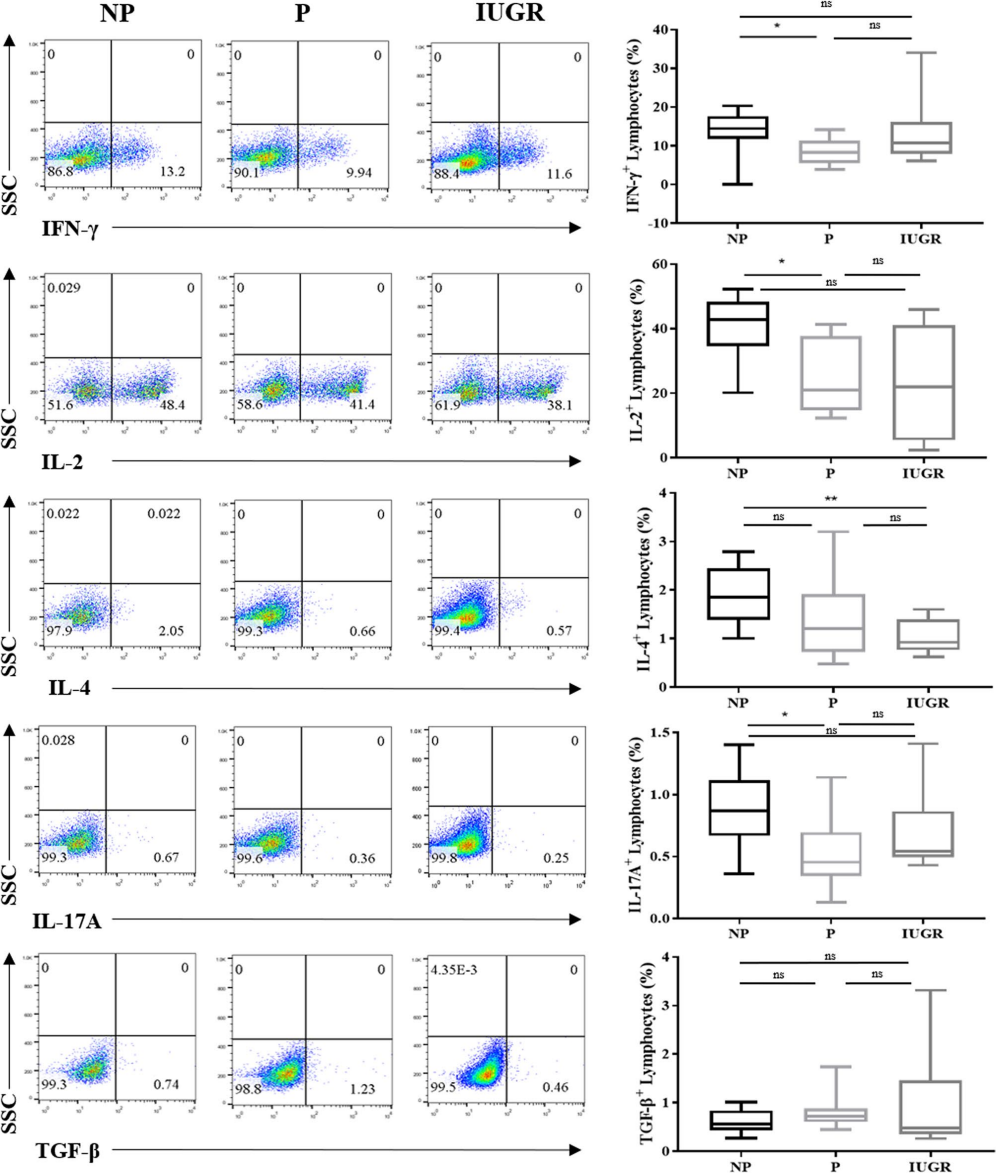
Th1 and Th17 cytokine production is altered in lymphocytes. Representative flow cytometry dot plots of cytokine production in response to PMA/Ionomycin stimulation in lymphocytes from NP, P and IUGR. Box and whisker plots indicate median and IQR of cells expressing IFN-γ, IL-2, IL-4, IL-17A, and TGF-β expression in lymphocytes from NP (n = 10), P (n = 9) and IUGR (n = 8). *p < 0.05, p** < 0.01, ns = non-significant, determined by Kruskal–Wallis with Dunn’s multiple comparisons test. Flow cytometry data were analysed using the FlowJo Software Version 10 (Becton Dickinson); website: https://www.flowjo.com/.

Our group and others have shown that there is a distinct suppression of Th1 and Th17 responses and increase in Th2 responses associated with normal pregnancy, and abrogation of these changes contributes to pathological pregnancies^24,31,44–48^. To corroborate these findings in our current cohort of patients, cytokine production was investigated in lymphocytes between NP, P and IUGR women by flow cytometry. The percentage of lymphocytes expressing pro-inflammatory cytokines were significantly reduced by ~ twofold in P compared to NP (IL-2: *p* = 0.04; IFN-γ: *p* = 0.03; and IL-17A: *p* = 0.04), but not in IUGR (Fig. 1). The lack of these changes during IUGR confer with previous reports where pregnancy success is characterised by reduced Th1/Th17 cytokine production and impairing this response contributes to pathological pregnancies including IUGR^44,45,49^. While there was no significant change in IL-4 levels in P relative to NP, a significant decrease was observed in IL-4 during IUGR compared to NP women in lymphocytes (*p* = 0.01) (Fig. 1).

### Th1 and Th17 are decreased during normal pregnancy, but not in IUGR

Our data shows the regulation of pro-inflammatory cytokines is significantly reduced in lymphocytes during normal pregnancies, however these changes were not observed in IUGR. Moreover, p65 levels in lymphocytes was decreased in P relative to NP, and increased in IUGR compared to P. These changes observed in lymphocytes prompted the question of whether p65 regulation impacts T-cell differentiation. To determine this, we first assessed transcription factor expression in CD4^+^ and CD8^+^ T-cells, and compared their expression between our cohorts.

**Figure 2:**
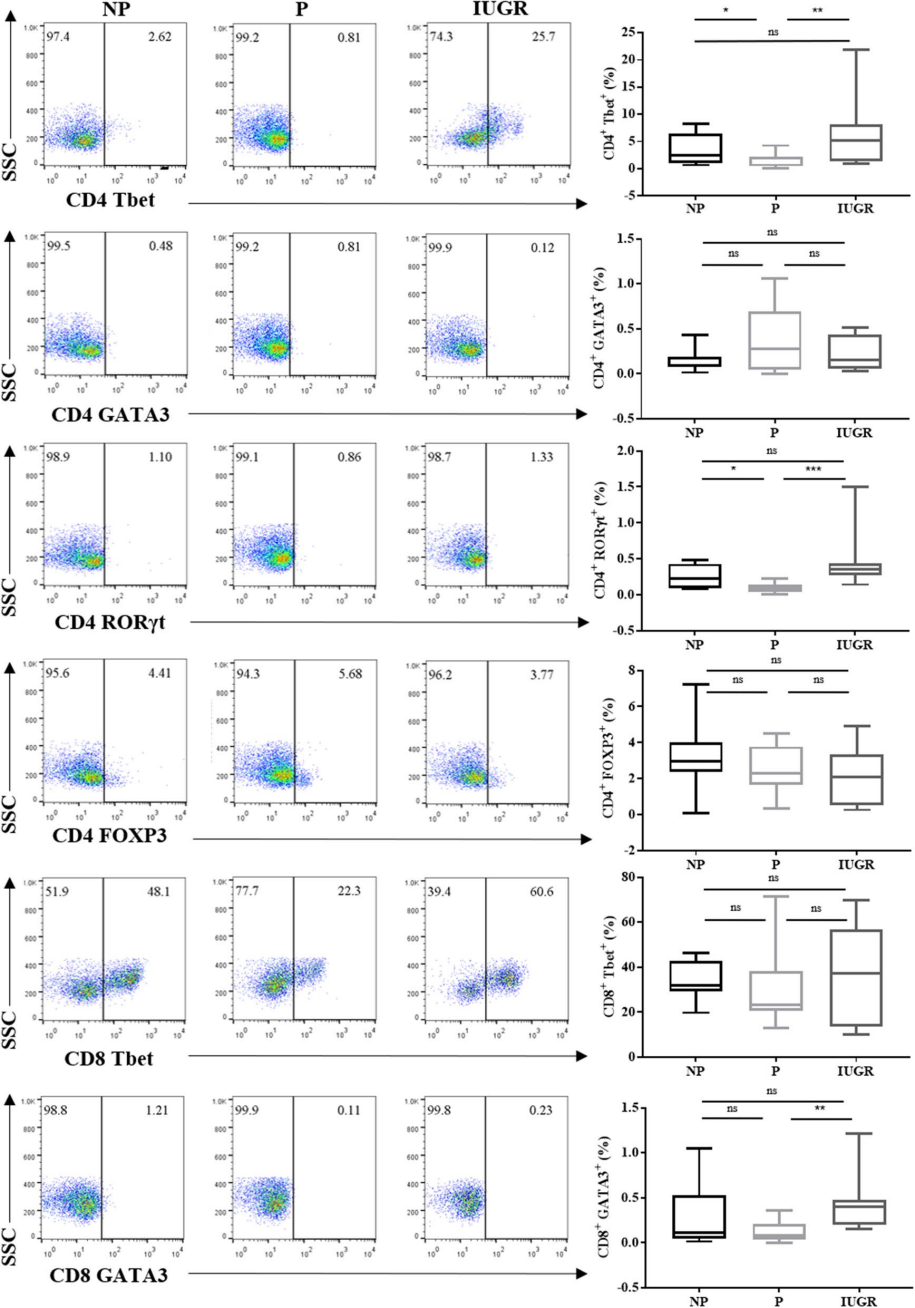
Tbet and RORγt is decreased in CD4^+^ T-cells during normal pregnancy compared IUGR. Representative flow cytometry dot plots of transcription factor expression in CD4^+^ Tbet (Th1), GATA3 (Th2), RORγt (Th17), FOXP3 (Treg) and CD8^+^ Tbet (Tc1), and CD8^+^ GATA3 (Tc2) T-cells from NP (n = 12), P (n = 12) and IUGR (n = 8). Box and whisker plots indicate median and IQR of T-cells expressing transcription factors. *p < 0.05, p** < 0.01, ns = non- significant, determined by Kruskal–Wallis with Dunn’s multiple comparisons test. Flow cytometry data were analysed using the FlowJo Software Version 10 (Becton Dickinson); website: https://www.flowjo.com/.

The basal profile of T-cell subsets in normal and IUGR pregnancies was determined (i.e. without PMA stimulation). As expression of transcription factors is not committed to the CD4 or CD8 lineage, but expressed by both^50^, T-cell subtypes were defined as: CD4^+^Tbet^+^ (Th1); CD8^+^Tbet^+^ Cytotoxic type 1 (Tc1); CD4^+^GATA3^+^ (Th2); CD8^+^GATA3^+^ (Tc2); CD4^+^RORγt^+^ (Th17); and CD4^+^FOXP3^+^ (Treg) (Fig. 2). Th1 and Th17 cells were significantly decreased in P compared to NP (Th1: *p* = 0.03; Th17: *p* = 0.005). Additionally, Th1 and Th17 cells in IUGR were significantly increased compared to P (Th1: *p* = 0.03; Th17: *p* = 0.001). Tc2 cells showed no difference in NP vs P but showed a significant increase in IUGR compared to P (*p* = 0.008). This suggests that pro-inflammatory helper T cell subsets Th1 and Th17 are dysregulated in IUGR compared to P.

### Expression of p65 in PBMC sub-populations is decreased in pregnancy compared to IUGR

**Figure 3:**
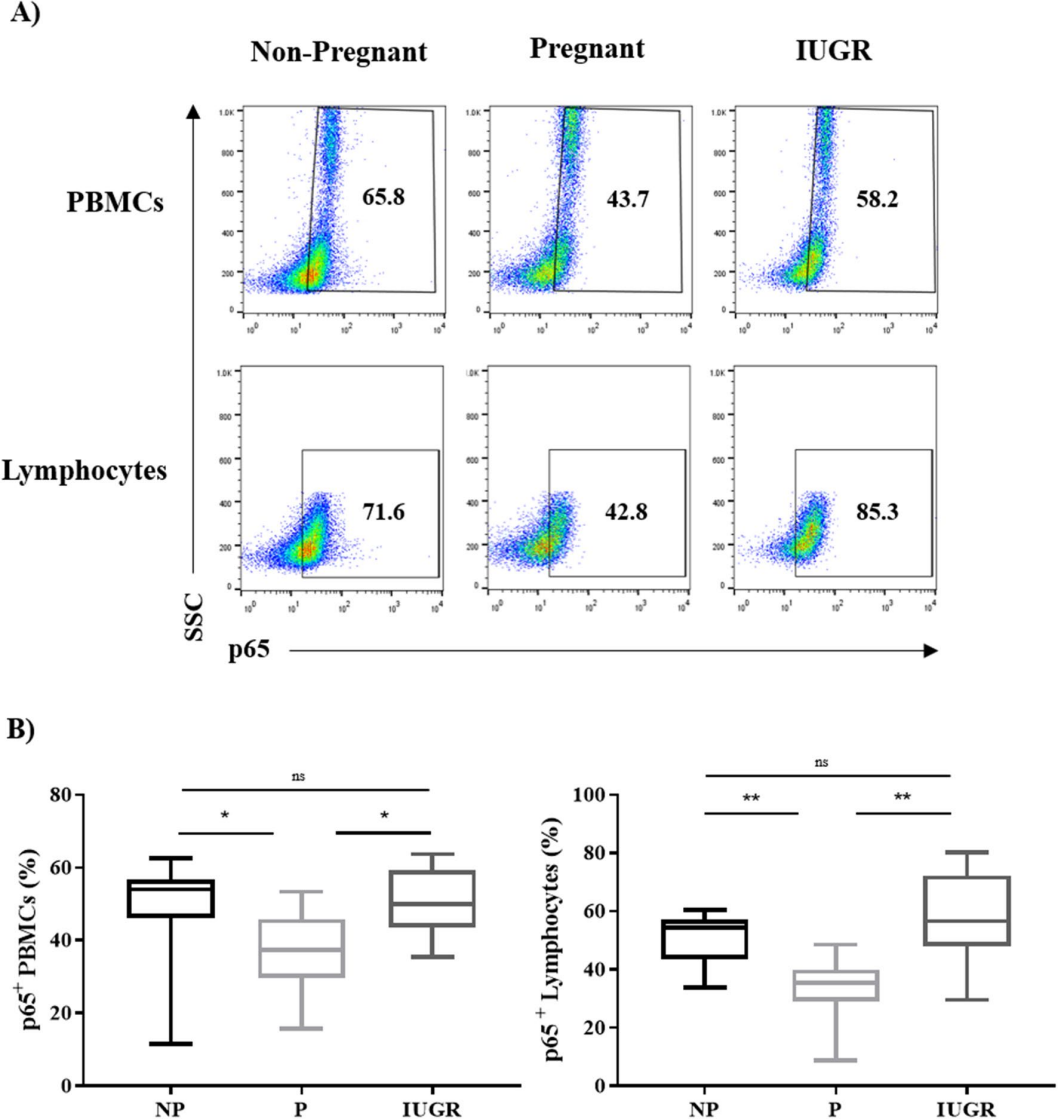
Expression of p65 in PBMCs and lymphocytes is reduced during normal pregnancy and increased during IUGR. (**A**) Representative flow cytometry dot plots of PBMCs and lymphocytes expressing p65 from NP (n = 11), P (n = 12) and IUGR women (n = 8). (**B**) Box and whisker plots indicate median and IQR of percentage of PBMCs and lymphocytes expressing p65. *p < 0.05, **p < 0.01, ns = non-significant, determined by Kruskal–Wallis with Dunn’s multiple comparisons test. Flow cytometry data were analysed using the FlowJo Software Version 10 (Becton Dickinson); website: https://www.flowjo.com/.

We have previously reported the suppression of p65 in maternal PBMCs relative to NP controls^30,51^, and have shown that p65 regulation alters cytokine production. Since aberrant cytokine production associates with IUGR pregnancies, we next determined whether p65 expression was also abnormal in IUGR, which would account for the changes in cytokine production in IUGR. Expression of p65 was measured as the percentage of cells expressing p65 in PBMCs and lymphocytes determined by the FSC and SSC profile (Fig. 3A). The percentage of p65^+^ cells was significantly decreased in PBMCs and lymphocytes of P compared to NP (*p* = 0.03 and *p* = 0.006 respectively) (Fig. 3B) consistent with our previously published data^51^. In contrast, there were significantly more p65^+^ PBMCs and lymphocytes in IUGR women compared to P women (*p* = 0.04 and *p* = 0.001 respectively), but no differences between IUGR and NP (Fig. 3B)*.* This suggests p65 is not suppressed in IUGR compared to P.

### Expression of p65 in T-cell subsets is altered in normal pregnancy and this is abrogated in IUGR

**Figure 4:**
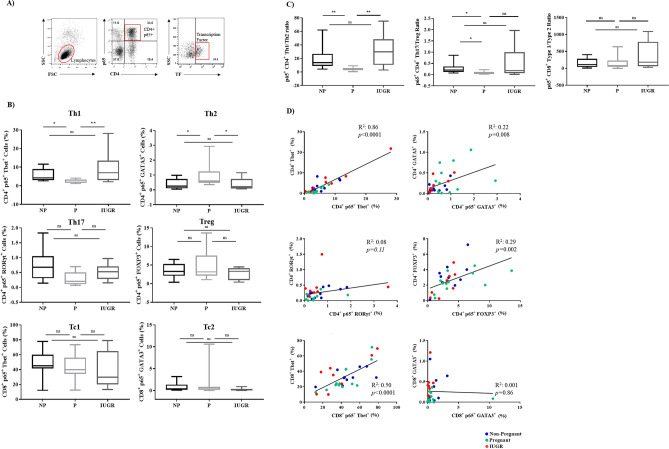
Expression of p65 in T-cell subsets is altered during normal pregnancy compared to IUGR. (**A**) Gating strategy for flow cytometry analysis of cells expressing p65 in CD4/CD8^+^ T-cells. (**B**) Percentage of T-cell subsets expressing p65 was determined by flow cytometry in Th1, Th2, Th17, Treg cells, Tc1, and Tc2 from NP (n = 11), P (n = 12) and IUGR (n = 8). Box and whisker plots indicate median and IQR of cells expressing p65. *p < 0.05, **p < 0.01*,* ns = non- significant, determined by Kruskal–Wallis with Dunn’s multiple comparisons test due to small sample size. (**C**) Percentage of cells expressing p65 was compared between pro-inflammatory and anti-inflammatory CD4^+^ T-helper cell subsets as a ratio of Th1 and Th2, or Th17 and Treg, or CD8^+^ Tc1 and Tc2, from non-pregnant women (NP) (n = 11), normal pregnant women (P) (n = 12) and pregnant women complicated with IUGR (n = 8). Box and whisker plots indicate median and IQR of ratios. *p < 0.05, **p < 0.01, ns = non-significant, according to the Kruskal–Wallis. (**D**) Linear regression analysis was used to assess the relationship between regulation of transcription factors and p65 expression in CD4^+^ and CD8^+^ T-cells. p65 expression was correlated with CD4^+^ Tbet (Th1), GATA3 (Th2), RORγt (Th17), FOXP3 (Treg), CD8^+^ Tbet (Tc1), or CD8^+^ GATA3 (Tc2). Non-Pregnant (n = 11; blue); Pregnant (n = 12; green); IUGR (n = 8; red). Flow cytometry data were analysed using the FlowJo Software Version 10 (Becton Dickinson); website: https://www.flowjo.com/.

To assess whether changes in p65 regulation underlie the observed changes in Th1 and Th17 subsets during normal and IUGR pregnancies, the proportion of p65^+^ T-cell subsets was assessed using the gating strategy outlined in Fig. 4A. Similar to the basal T-cell subsets, percentage of p65^+^ Th1 cells was decreased in P when compared to NP (*p* = 0.04), but not IUGR (*p* = 0.008; Fig. 4B). A similar trend was observed for p65^+^ Th17 cells, though this did not reach statistical significance. In contrast to the basal profiles, a significant increase in the percentage of p65^+^ Th2 cells was noted in P compared to NP (*p* = 0.03), but this elevation was impaired in IUGR (*p* = 0.04). The change in T-cell phenotypes was specific to p65^+^ cells as there were no differences in individual populations of T-cells in p65^low^ cells (Supplementary Fig. 1). This suggests that p65 is dysregulated in helper T cell subsets in IUGR compared to P.

In pregnancy, the balance between pro- and anti-inflammatory T-cell subsets is important for maintaining pregnancy success. As cytokines promote the differentiation of one subset and inhibit the differentiation of the opposing subset^52–57^, we assessed the ratios of p65^+^ Th1/Th2, Th17/Treg, and Tc1/Tc2 (Fig. 4C). This analysis showed that the p65^+^ Th1/Th2 ratio was ~ fourfold lower in P than NP (*p* = 0.003), and ~ ninefold higher in IUGR compared to P (*p* = 0.003). Similarly, the Th17/Treg ratio was threefold lower in P compared to NP (*p* = 0.01). However, no statistically significant differences in the Th17/Treg ratio between IUGR and P was observed (*p* = 0.09). Despite CD4^+^ and CD8^+^ T-cells lineages sharing the same transcriptional factors during development, there was no statistical differences in the p65^+^ Tc1/Tc2 ratio. This suggests that a failure to suppress p65 in Th1 and Th17 cells in IUGR pregnancies may contribute to the pathology.

Combined, these data led to the investigation of whether there was a correlation between the percentage of T-cell subsets as indicated by transcriptional factor expression (CD4^+^ Tbet^+^;) and the percentage of p65^+^ T-cells from each subset (i.e. CD4^+^ p65^+^ Tbet^+^). To determine this, linear regression analysis was performed between transcription factor expression, and p65 expression in T-cell subsets (Fig. 4D). Tbet expression was significantly associated with p65^+^ expression in both CD4^+^ (R^2^ = 0.86, *p* < 0.0001) and CD8^+^ T-cells in all patients (R^2^ = 0.5, *p* < 0.0001). Significant correlation was also seen in CD4^+^ T-cells between p65 expression and GATA3 (R^2^ = 0.22, *p* = 0.008), and FOXP3 (R^2^ = 0.29, *p* = 0.002). However, significance in both cases showed a low R-squared value, demonstrating high levels of variability which may affect the true precision of this prediction. There was no significant correlation between p65 expression and RORγt expression in CD4^+^ T-cells or GATA3 expression in CD8^+^ T-cells. These findings collectively demonstrate that CD4^+^ Th1 and CD8^+^ Tc1 differentiation through the transcriptional regulation of Tbet, are dependent on the expression of p65. Thus, this highlights the requirement for strict p65 regulation in pregnancy to provide the environment for pregnancy success.

### Fas expression in maternal immune cells is unchanged during pregnancy

While the mechanism that regulates maternal T-cell p65 suppression is not established, Fas activation is reported to regulate suppression of p65 via caspase degradation^35^. In Jurkat T-cells, we and others have shown that specific Fas activation, and FasL^+^ EVs degrades p65 and the T-cell receptor transmembrane protein CD3ζ, both of which are integral for T-cell survival and function^37,38^. Therefore, Fas expression was assessed in P, NP and IUGR women and we showed no significant difference in expression within PBMCs, lymphocytes, CD4^+^ or CD8^+^ T-cells between any of the patient cohorts (Supplementary Fig. 2).

### Healthy maternal plasma alters p65^+^ T-cell subsets

**Figure 5:**
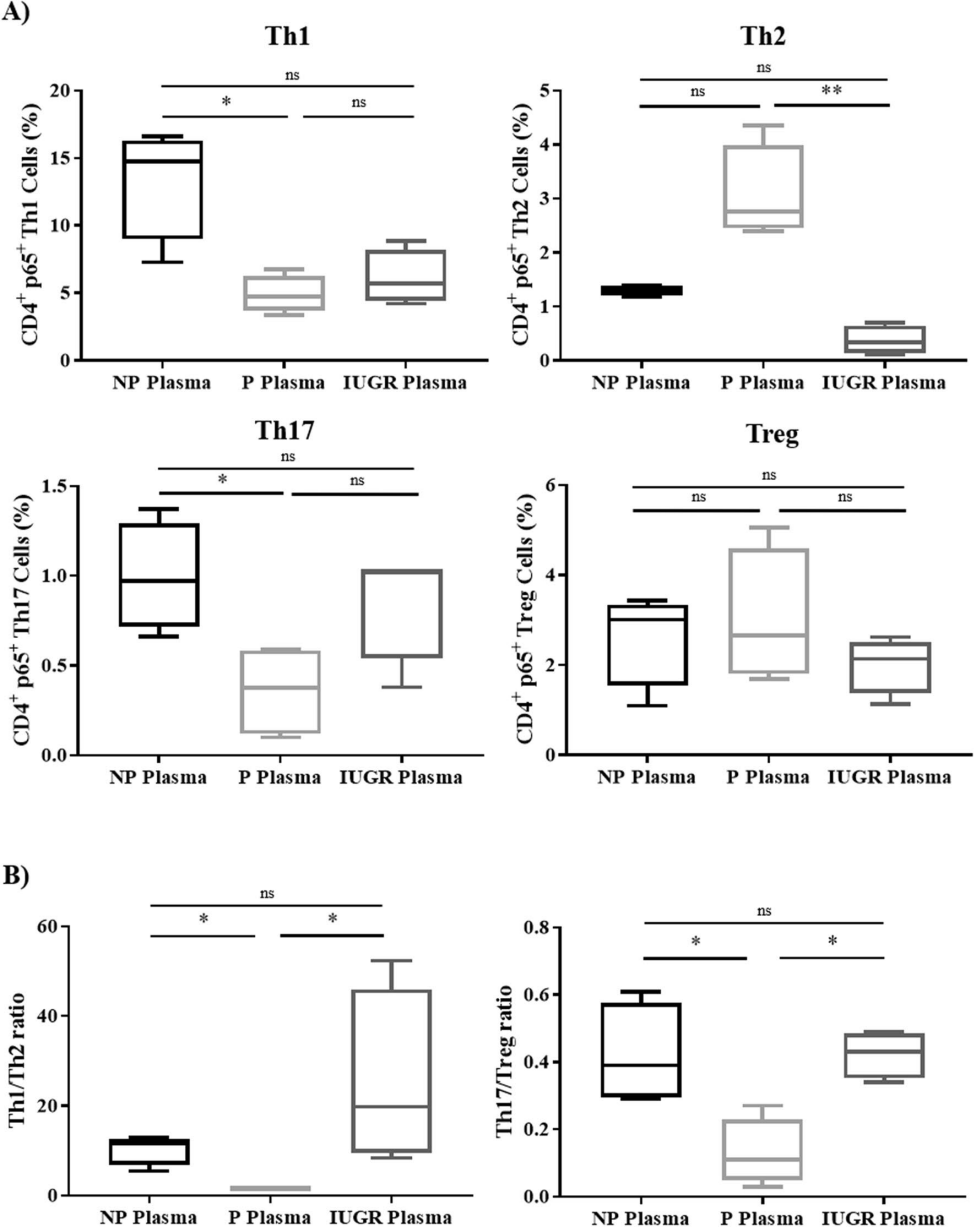
Expression of p65 in T-cell subsets is altered in the presence of healthy maternal plasma. Non-pregnant PBMCs were cultured in the presence of 20% NP, P or IUGR plasma (all n = 4). (**A**) Percentage of cells expressing p65 was determined by flow cytometry in CD4^+^ Th1, Th2, Th17 and Treg cells. (**B**) Percentage of cells expressing NF-κB p65 compared between pro- and anti-inflammatory cell types as a ratio between Th1 and Th2, or Th17 and Treg. Box and whisker plots indicate median and IQR of cells expressing p65. *p < 0.05, **p < 0.01, ns = non-significant, determined by Kruskal–Wallis with Dunn’s multiple comparisons test. Flow cytometry data were analysed using the FlowJo Software Version 10 (Becton Dickinson); website: https://www.flowjo.com/.

We have previously shown p65 expression in PBMCs is altered by maternal plasma whereby isolated maternal EVs suppress T-cell p65 more than EVs from NP plasma, and the removal of EVs from maternal plasma post ultra-centrifugation prevents down regulation of p65 in T cells^37^. While Fas expression by P and IUGR T cells was also unchanged, we next assessed whether the source of p65 suppression was attributed to EVs in the maternal plasma^37,38^ and which T cell subsets are modulated. Maternal plasma and PBMCs from non-pregnant women were isolated and cultured with 20% (v/v) plasma from non-pregnant, pregnant and IUGR pregnant women for 72 h and assessed by flow cytometry. The number of p65^+^ Th1 and Th17 cells significantly decreased when PBMCs from NP women were cultured in the presence of P plasma compared to NP plasma (Th1: *p* = 0.03; Th17: *p* = 0.03) (Fig. 5A). In contrast, no significant difference in p65^+^ Th1 or Th17 cells when PBMCs from NP women were cultured in IUGR plasma compared to P plasma. Additionally, there were no significant differences in the T-cell subtypes when PBMCs from NP women were cultured in the presence of plasma from IUGR pregnancies when compared to plasma from NP women. IUGR plasma significantly decreased the amount of p65^+^ Th2 cells in PBMCs from NP women compared to P plasma (*p* = 0.005) (Fig. 5A). In addition to altering the T-cell phenotype, maternal plasma was capable of altering the Th1/Th2 and Th17/Treg ratios. There was a significant ~ tenfold decrease in the p65^+^ Th1/Th2 ratio in PBMCs from NP women in response to P plasma compared to NP plasma (*p* = 0.02), and a ~ 15-fold increase in response to IUGR plasma compared to P plasma (*p* = 0.02) (Fig. 5B). Similarly, the p65^+^ Th1/Th17 ratio decreased in response to P plasma compared to NP plasma (*p* = 0.03) and was significantly increased in response to IUGR plasma compared to P plasma (*p* = 0.03) (Fig. 5B). These results indicate that P plasma has the ability to regulate T-cell phenotypes, but plasma from IUGR pregnancies has a limited capacity to regulate p65 expression and subsequent T-cell phenotypes. This suggests that the factor present in plasma of normal pregnancies is absent or defective in plasma of IUGR pregnancies, and that the EV phenotype may differ in pathological pregnancies.

### Plasma derived vesicles is reduced in IUGR plasma compared to normal pregnancies

To clarify whether there are differences in plasma derived EVs during normal and IUGR pregnancies, the number of EVs was quantified in samples of plasma from NP, P, and IUGR pregnancies.

**Figure 6:**
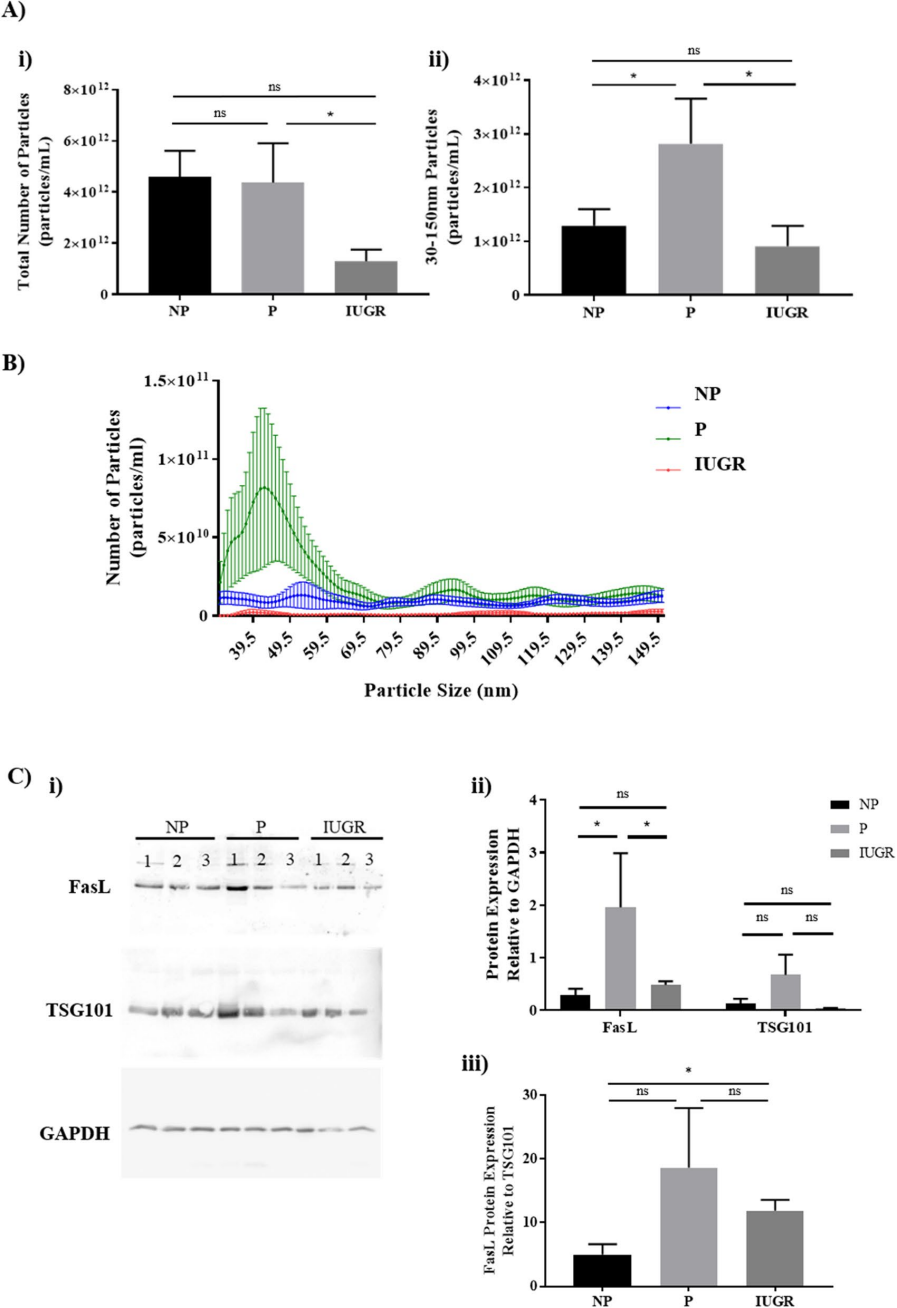
Particle concentration from plasma is reduced during IUGR compared to normal pregnancies. Nanoparticle tracking analysis (NTA) was used to quantify EVs present in plasma of NP, P, IUGR (all n = 8). (**A**) Bar graphs represent the median and IQR of total number of particles, and particles between 30–150 nm. (**B**) Line graph represents the mean ± SEM of the particles between 30–150 nm exosomes. Profile for NP (▬), P(▬) and IUGR (▬) plasma is shown. *p < 0.05, determined by Kruskal–Wallis with Dunn’s multiple comparisons test. (**C**) EVs isolated from pregnant plasma were characterised by exosomal marker TSG101, and FasL expression in NP, P and IUGR samples. (i) Blots are representative of 3 patients per cohort. Full length blots are presented in Supplementary Fig. 3, where gel edges that are not evident in the uncropped GAPDH gel are indicated by the digital image of the gel. (ii) Bars indicate median and IQR of densitometric analysis of FasL and TSG101 protein expression as a fold change relative to house keeping protein GAPDH from 4 patients per cohort. (iii) Bars indicate median and IQR of densitometric analysis of FasL protein expression as a fold change relative to TSG101 from 4 patients per cohort. *p < 0.05, ns = non-significant, determined by Mann–Whitney due to a small sample size. Densitometry of western blots was performed using the Image-J digital software version 1.8.0; website: https://imagej.nih.gov/ij/. Particle concentration and size distribution were automatically analysed with the NanoSight NTA Software Version 3.2 (Malvern Instruments, UK).

Although there was no significant difference in the total number of EVs in P or IUGR plasma compared to NP plasma (Fig. 6A i), IUGR plasma contained less EVs than P plasma (*p* = 0.03) (Fig. 6A i). In addition, exosomes with a size range of 30–150 nm were significantly increased in P plasma relative to NP and IUGR plasma (*p* = 0.04 and *p* = 0.03; Fig. 6A ii and 6B). Isolated plasma exosomes were further characterised by western blot analysis to determine FasL expression. The presence of exosomes was confirmed by TSG101 expression (Fig. 6C). FasL expression was significantly elevated by ~ fourfold in P but not IUGR women compared to NP women (*p* = 0.02; Fig. 6C ii), while TSG101 expression did not reach statistical significance. Moreover exosomes from IUGR pregnancies showed significantly lower FasL protein expression compared to P controls (*p* = 0.03; Fig. 6C ii). To determine whether reduced FasL expression was attributable to reduced TSG101^+^ exosomes, FasL expression was normalized to TSG101 and showed that despite significantly fewer exosomes in IUGR compared to P (Fig. 6A ii), FasL/TSG101 expression was comparable to P, and greater than NP (Fig. 6C iii). Therein lack of sufficient FasL^+^ exosomes in maternal plasma may underlie the abrogated regulation of T-cell differentiation in IUGR pregnancies.

## Discussion

The maternal immune system plays a critical role for the maintenance of successful pregnancy, yet the mechanisms that control this are not fully understood. In this study, we demonstrate that suppression of Th1 and Th17 cytokines during normal pregnancy is attenuated in pregnancies complicated with IUGR, and that suppression of NF-κB, a molecule notable for regulating various aspects of T-cell immunity is similarly attenuated. Moreover, we have shown a role for FasL^+^ exosomes in regulating p65 in T-cells, and provide the first evidence that abnormal exosome production may underlie abnormal immunity associated with IUGR. Studies suggest that the maintenance of pregnancy is mediated by decreased Th1 and Th17 cytokine production^24,44–48^ and an overproduction of Th1 and Th17 cytokines contributes to the pathogenesis of preeclampsia, recurrent pregnancy losses and implantation failure^45,46,48,58–60^. In this study, we showed that Th1 cytokines IL-2 and IFN-γ were significantly decreased in PMA-stimulated lymphocytes from women in the third trimester of pregnancy, compared to NP controls. Of pertinence, was the lack of these changes during IUGR. These findings corroborate with previous reports where pregnancy success is characterised by reduced Th1/Th17 cytokine production^44,45,49^, and impairing this response contributes to IUGR. The suppressed cytokine environment in pregnancy correlated with a suppression of p65 in PBMCs and lymphocytes. This implies that in pregnancy, rather than complete inhibition of p65, a critical level of suppression is reached to see an effect in maternal T-cells. This is perhaps a critical mechanism for tolerance towards fetal antigens, ultimately preventing pathological complications associated with pregnancy. This notion is supported by a report showing that the magnitude of phosphorylation of p65 governs the degree of response in NK cells^61^, which could also be true of T-cells. Interestingly, 2/8 women with IUGR recruited for this study, were on Betamethasone which is prescribed to lessen the risk of the birth of a severely premature infant. Despite these patients being treated with an immunosuppressive drug, there was still a difference in the cytokine profile between normal and IUGR pathological pregnancies. Overall, the inflammatory cytokine environment associated with IUGR also correlated with a lack of p65 suppression in PBMCs and lymphocytes, indicating a potential mechanism by the placenta to dictate the maternal immune environment during IUGR.

Given that there were key differences in p65 expression between normal and IUGR pregnancies in PBMCs and lymphocytes which correlated with a change in cytokine profile, another objective of this study was to determine which cell types showed reduction in p65 in pregnancy, and whether this is impaired during IUGR. We observed a significant decrease in the absolute number of p65^+^ Th1 cells during normal pregnancy compared to NP women, which was consistent with reduced production of Th1 cytokines in response to activation. This data concurs with previous reports demonstrating the link between NF-κB and Th1 cytokine production^62^, and previous data published from our group, showing inhibition of NF-κB translocation results in reduced Th1 cytokine production in NP T-cells^30^. Moreover, our study also showed a clear correlation between p65 and Tbet expression thus highlighting the preferential role of this pathway in mediating Th1 immune responses. Despite a reduction in IL-17A production observed in this study, there was no reduction in p65^+^ Th17 cells. Evidence suggests that p65 is involved in Th17 differentiation and p65-deficient T-cells have impaired IL-17 gene expression and Th17 cell differentiation^34^; however, activation of p65 is no longer required once the cells are polarised to a Th17 phenotype—indicating its role in differentiation, but not lineage specific-function. In addition to observing a decrease in p65^+^ Th1^+^ cells during pregnancy, an increase in p65^+^ Th2 cells was also observed. As evidences shows that p50 regulates GATA3 expression, but not p65^63^, this increase may reflect the lack of p65 activation in polarised Th2 cells which was not assessed in this study. Moreover, the lack of changes in p65^+^ FOXP3^+^ cells suggests that transcriptional control of Treg cells may not be regulated by p65 during pregnancy. However, it has been previously shown that there is a direct Treg-lineage specific role for NF-κB p65 and Treg-associated genes, such as FOXP3^64–66^. Despite our results not showing a direct impact in p65^+^ FOXP3 cells during pregnancy, our data did show a positive correlation between p65 and FOXP3 with high significance, suggesting a potential role for Treg regulation through p65. But it is also possible that other canonical NF-κB subunits may regulate lineage specific transcription factor for Tregs such as FOXP3. This is supported by other evidence showing that c-Rel rather than p65 plays a more important role in Treg cell differentiation by enhancing FOXP3 expression^67,68^. Moreover, the correlation between p65 expression with a reduction in the ratio between Th1/Th2 and Th17/Treg immune responses during pregnancy, which was not observed during IUGR, suggests that lymphocytes are endogenously polarised towards a Th1 and Th17 pro-inflammatory profile during IUGR, and for Th1, this is mediated through dysregulation of p65.

Currently, the mechanisms or source that control the necessary suppression of p65 for pregnancy success have not been determined. We have previously shown that Fas activation suppresses p65 expression in Jurkat T-cells, which is reversed with the Fas inactivating antibody ZB4^37^. Furthermore, Fas expression is elevated in T-cells during pregnancy^47^, which suggests a possible mechanism to eliminate T-cells reactive against fetal antigens. This is in stark contrast to the findings from this study, as no significant differences in Fas expression were observed in PBMCs, lymphocytes or T-cells during pregnancy under basal conditions. An explanation for the lack of elevated Fas expression in pregnancy may be a mechanism to resist excessive apoptosis and loss of T-cells, as maternal T-cells are also required to mount immune responses against microbial and intracellular pathogens, not just immune tolerance of the fetus. Thus, it is speculated that the availability of Fas expression does not dictate the regulation of p65 during pregnancy. As the downregulation of p65 is pregnancy specific, and the placenta plays a vital role in cross talk between mother and fetus, it is possible that factors produced by the placenta express FasL and are capable of affecting p65 regulation, contributing to the changes seen in maternal T-cell responses during pregnancy.

Cell communication is a common mechanism that regulates cellular homeostasis. Well defined mechanisms of intercellular communication consist of: 1) soluble mediators such as hormones, cytokines and chemokines acting in an endocrine manner; 2) direct adhesion between cells; and 3) transfer of information through nanotubules^69,70^. More recently, exosomes have been recognised as potent facilitators of intercellular communication^71^. In our study, we show that total number of exosomes (characterised with a diameter of 30–150 nm) from plasma of IUGR pregnancies were significantly reduced relative to normal pregnancies. While the role of exosomes during normal pregnancy and pathological pregnancies, specifically IUGR, is not well studied, the total concentration of circulating exosomes was significantly decreased between IUGR cases and healthy maternal controls^72^, which is consistent with our findings. This lack of exosome production is likely a consequence of the placental insufficiency associated with IUGR^73,74^. Furthermore, the lack of FasL expression on exosomes from IUGR suggests that abrogated Fas/FasL signaling may underly the cause of increased inflammation associated with IUGR, which the data from this study suggests is through the dysregulation of p65 expression. This suggests that the placenta is directing T-cells towards appropriate phenotypes for pregnancy success through the production of exosomes, identifying a functional role for exosomes in regulating p65 during pregnancy.

In conclusion, our study demonstrates that normal pregnancy is associated with the suppression of p65 and a corresponding reduction in pro-inflammatory cytokine production. This contributes to a reduction in the Th1/Th2 and Th17/Treg immune cell profile, and is altered in pregnancies affected by IUGR. Our findings extend our knowledge in the role of exosomes during pregnancy and suggests that FasL^+^ placenta derived exosomes may execute a program of maternal immune adaptation from the maternal–fetal interface into the peripheral circulation. The lack of sufficient FasL^+^ exosomes in maternal plasma of IUGR pregnancies together with the inability of maternal plasma to reduce inflammatory responses, imply that exosomes are key to regulating appropriate maternal responses for successful pregnancy. Further investigation exploring the qualitative properties of exosomes, as well as placental production of exosomes during IUGR, may shed more light on their role in modulating maternal immunity.

## Supplementary Information

Supplementary Information.
