# Supplementary material for: NF-κB regulation in maternal immunity during normal and IUGR pregnancies
Source: Sci Rep. 2021 Oct 25;11:20971. doi: 10.1038/s41598-021-00430-3 (PMC8545974; doi:10.1038/s41598-021-00430-3)
Supplement: Supplementary file 1 — Supplementary Information. [file 41598_2021_430_MOESM1_ESM.docx]

Supplementary Information

Table 1. Surface and Intracellular Antibodies Per Test for Flow Cytometry

| **Antibody** | **Clone** | **Catalogue**  **Number** | **Company** | **Volume per test (µl)** |
| --- | --- | --- | --- | --- |
| CD4 FITC | L200 | 550628 | BD Pharmingen | 3 |
| CD8 FITC | RPA-T8 | 561948 | BD Pharmingen | 3 |
| CD95 PECy5 | DX2 | 559773 | BD Pharmingen | 3 |
| Tbet AF647 | 4B10 | 561264 | BD Pharmingen | 1 |
| GATA3 AF647 | L50-823 | 560068 | BD Pharmingen | 5 |
| RORγt AF647 | Q21-559 | 56320 | BD Pharmingen | 1 |
| FOXP3 AF647 | 259D/C7 | 560045 | BD Pharmingen | 5 |
| IFN-γ PE | B27 | 562016 | BD Pharmingen | 5 |
| IL-2 PE | MQ1-17H12 | 560902 | BD Pharmingen | 5 |
| IL-4 PE | 8D4-8 | 559333 | BD Pharmingen | 5 |
| IL-17A PE | SCPL1362 | 560346 | BD Pharmingen | 5 |
| TGF-β PE | FAB241P | 25508 | R&D Systems | 1 |
| NF-κB p65 PE | 14G10A21 | 653004 | BioLegend | 2.5 |

Table 2. Antibodies for Western Blotting

| **Antibody** | **Clone** | **Catalogue Number** | **Company** | **Dilution Factor** |
| --- | --- | --- | --- | --- |
| Rabbit Anti-Human FasL | N-20 | sc-834 | Santa Cruz, USA | 1 in 1000 |
| Mouse Anti-Human TSG101 | C-2 | sc-7964 | Santa Cruz, USA | 1 in 500 |
| Mouse Anti-Human GAPDH | O411 | sc-47724 | Bio-Rad, USA | 1 in 1000 |
| Goat Anti-Rabbit IgG (HRP) |  | 170-6515 | Bio-Rad, USA | 1 in 1000 |
| Goat Anti-Mouse IgG (HRP) |  | 170-6516 | Bio-Rad, USA | 1 in 1000 |

Supplementary Figure 1.

***Supplementary Figure 1. Percentage of CD4^+^ and CD8^+^ T-cell effector subsets expressing p65^low^ is unchanged during pregnancy.*** Percentage of T-cell subsets expressing p65^low^ was determined by flow cytometry in CD4^+^ and CD8^+^ T-cells, and effector subsets including Th1, Th2, Th17, Treg cells, Tc1, and Tc2 from NP (n=11), P (n=12) and IUGR (n=8). Box and whisker plots indicate median and IQR of cells expressing p65. **p*<0.05, **p<0.01, ns = non- significant, determined by Kruskal-Wallis with Dunn’s multiple comparisons test due to small sample size. Flow cytometry data were analysed using the FlowJo Software Version 10 (Becton Dickinson); website: https://www.flowjo.com/.


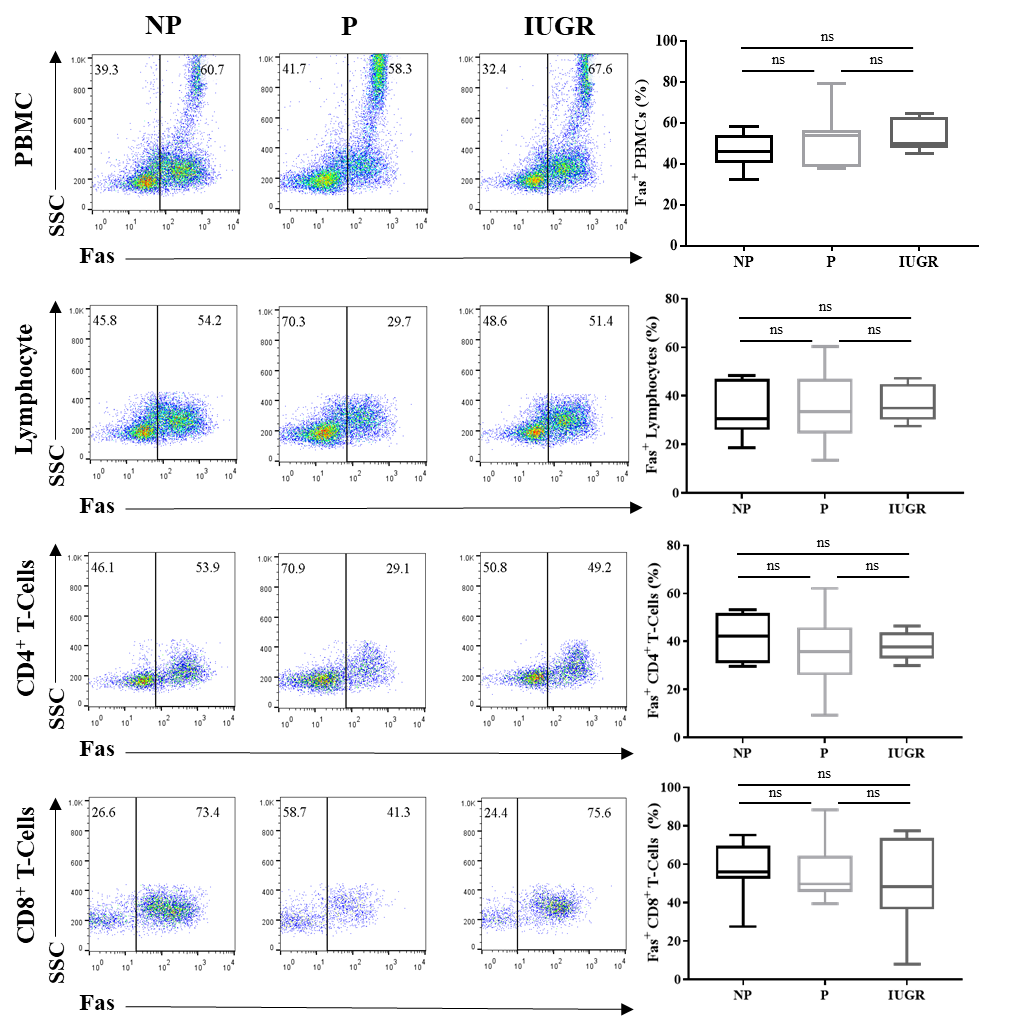
Supplementary Figure 2.

***Supplementary Figure 2. Percentage of PBMCs, lymphocytes, CD4^+^ and CD8^+^ T-cells expressing Fas is unchanged during pregnancy.*** Percentage of cells expressing Fas was determined by flow cytometry in PBMCs, lymphocytes, CD4^+^ and CD8^+^ T-cells from non-pregnant women (NP) (n=11), normal pregnant women (P) (n=12) and pregnant women complicated with IUGR (n=8). Box and whisker plots indicate median and IQR of PBMCs, lymphocytes and T-cells expressing Fas. ns = non-significant, determined by Kruskal-Wallis with Dunn’s multiple comparisons test. Flow cytometry data were analysed using the FlowJo Software Version 10 (Becton Dickinson); website: https://www.flowjo.com/.

**Supplementary Figure 3.**

***
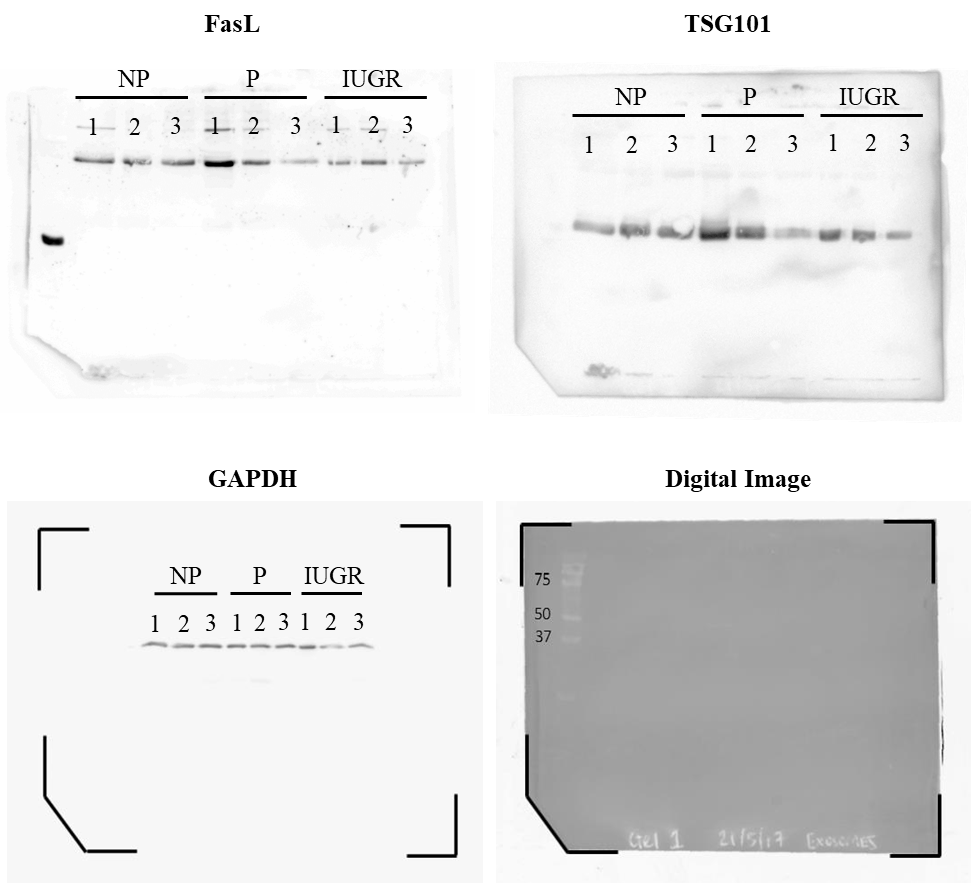
***

***Supplementary Figure 3. FasL and TSG101 protein expression of plasma from IUGR pregnancies is altered compared to normal pregnancies.*** Exosomes were characterised by western blot analysis of FasL and TSG101 protein expression in plasma from non-pregnant (NP), pregnant (P), and IUGR women, relative to the house keeping protein GAPDH. Blot shows 3 patients per group, from a total of 4 patients per cohort. Digital image of gel indicates membrane edges, and protein molecular weight marker in kDa. Densitometry of resulting blots was performed using the Image-J digital software version 1.8.0; website: https://imagej.nih.gov/ij/.
